# Detection of Specific ZIKV IgM in Travelers Using a Multiplexed Flavivirus Microsphere Immunoassay

**DOI:** 10.3390/v10050253

**Published:** 2018-05-12

**Authors:** Carmel T. Taylor, Ian M. Mackay, Jamie L. McMahon, Sarah L. Wheatley, Peter R. Moore, Mitchell J. Finger, Glen R. Hewitson, Frederick A. Moore

**Affiliations:** Public Health Virology, Forensic and Scientific Services, Queensland Health, Coopers Plains, Queensland, 4108, Australia; ian.mackay@health.qld.gov.au (I.M.M.); Jamie.McMahon@health.qld.gov.au (J.L.M.); Sarah.Wheatley@health.qld.gov.au (S.L.W.); Peter.Moore2@health.qld.gov.au (P.R.M.); Mitchell.Finger@health.qld.gov.au (M.J.F.); Glen.Hewitson@health.qld.gov.au (G.R.H.); Frederick.Moore@health.qld.gov.au (F.A.M.)

**Keywords:** Zika virus, serology, flavivirus, microsphere immunoassay, validated, optimised, dengue virus

## Abstract

Zika virus (ZIKV) has spread widely in the Pacific and recently throughout the Americas. Unless detected by RT-PCR, confirming an acute ZIKV infection can be challenging. We developed and validated a multiplexed flavivirus immunoglobulin M (IgM) microsphere immunoassay (flaviMIA) which can differentiate ZIKV-specific IgM from that due to other flavivirus infections in humans. The flaviMIA bound 12 inactivated flavivirus antigens, including those from ZIKV and yellow fever virus (YFV), to distinct anti-flavivirus antibody coupled beads. These beads were used to interrogate sera from patients with suspected ZIKV infection following travel to relevant countries. FlaviMIA results were validated by comparison to the ZIKV plaque reduction neutralization test (PRNT). The results highlight the complexity of serological ZIKV diagnosis, particularly in patients previously exposed to, or vaccinated against, other flaviviruses. We confirmed 99 patients with ZIKV infection by a combination of RT-PCR and serology. Importantly, ZIKV antibodies could be discriminated from those ascribed to other flavivirus infections. Serological results were sometimes confounded by the presence of pre-existing antibodies attributed to previous flavivirus infection or vaccination. Where RT-PCR results were negative, testing of appropriately timed paired sera was necessary to demonstrate seroconversion or differentiation of recent from past infection with or exposure to ZIKV.

## 1. Introduction

Zika virus (ZIKV) is an arthropod-borne virus (arbovirus) assigned to the genus *Flavivirus* of the family *Flaviviridae*. The virus was identified in 1947 in Uganda [1]. Following outbreaks in western Pacific and southern Pacific regions, ZIKV has been grouped into three genetically distinct lineages, East African, West African and Asian. Most infections have been reported to be asymptomatic [2]. When present, signs and symptoms are nonspecific and overlap with those due to other often co-circulating arboviruses including dengue virus (DENV) [3]. A suspected human case of ZIKV disease may include rash, fever, conjunctivitis, arthralgia, arthritis, myalgia, headache, malaise and fatigue [4]. After an outbreak in French Polynesia in 2013–2014, neurological complications and congenital cerebral malformations were identified [3,5]. From 2015, a relationship between ZIKV, congenital infection and central nervous system malformation and disease became known as congenital Zika virus syndrome [6,7,8]. Where resources permit, PCR-based molecular diagnostics are used to support clinical decision-making by detecting acute infection. Molecular methods to detect arboviruses are less effective when samples have low viral loads such as occurs during the short-lived viremia caused by ZIKV. Serology extends the infection identification window but is often cross-reactive; that is, antibodies triggered by one flavivirus infection may react with various other flaviviruses. In some instances, an extended panel of serological virus testing may assist in getting a specific diagnosis.

We previously described an IgM capture ELISA (MAC-ELISA) which could sensitively and specifically differentiate between infections due to any of the four DENV serotypes, Japanese encephalitis virus (JEV), Murray Valley encephalitis virus (MVEV), Kunjin virus (KUNV), Alfuy virus (ALFV), Kokobera (KOKV) and Stratford virus (STRV) [9]. We subsequently developed a novel multiplexed flavivirus IgM typing microsphere immunoassay (flaviMIA) to replace the MAC-ELISA, with the inclusion of YFV and eventually, in response to the sudden outbreak across the South Pacific region, ZIKV. We use the flaviMIA diagnostic assay to differentiate IgM antibodies to locally acquired and commonly imported flaviviruses [10].

We describe the development, validation, challenges and application of the flaviMIA consisting of a panel of 12 flaviviruses which are either endemic or relevant to travelers visiting or returning to Australia. The flaviMIA allows identification of plaque reduction neutralization test (PRNT)-confirmed ZIKV-specific IgM among sera sent to a public health virology laboratory for characterization.

## 2. Materials and Methods

### 2.1. Bead Coupling to Monoclonal Antibody for FlaviMIA

Bio-Plex Pro Magnetic carboxylated beads (Bio-Rad, Hercules, CA, USA) were coupled to the 6B6C-1 flavivirus monoclonal antibody [11] using a Bio-Plex Amine Coupling Kit (Bio-Rad, USA). Generally, 1.25 × 10^6^ beads were coupled to approximately 10–12 µg of antibody. The antibody was either purified in-house from stocks of murine ascitic fluid, or obtained commercially (Millipore, Burlington, MA, USA). Coupled microspheres were stored at 4 °C at approximately 10^7^ beads per mL.

### 2.2. Production of Purified Whole Virus Antigen Preparations for FlaviMIA

ZIKV strain MR766 was sourced from the American Type Culture Collection (Manassas, VA, USA). This was the only strain available at short notice permitting us to rapidly develop tests in response to a public health need. The literature suggests there is only a single serotype of ZIKV and there is no evidence that different phylogenetic lineages react differently to IgG and IgM [12]. The vaccine strain of YFV (17D) was used to prepare YFV antigen. Other flaviviruses (DENV serotypes 1–4, JEV, MVEV, KUNV, ALFV, KOKV and STRV) were as described previously [9,13]. ZIKV was cultured in the C6/36 mosquito cell line for seven days in Gibco Opti-MEM Reduced Serum Medium (Thermo Fisher Scientific, Waltham, MA) + 0.2% Bovine Serum Albumin Fraction V (Thermo Fisher Scientific, Waltham, MA, USA) followed by virus inactivation for 24 h using binary ethyleneimine (BEI) prepared from 2-Bromoethylamine hydrobromide (Sigma-Aldrich, St. Louis, MO, USA [14]). ZIKV was concentrated from the supernatant using Amicon Ultra 3 kDa centrifugal filters (Millipore, USA), following which the viral inactivation was confirmed by three serial passages in C6/36 cells followed by immunofluorescence testing using a panel of monoclonal antibodies. The other flaviviruses were prepared by cross-flow filtration and inactivated as previously described [14]. Where required, the preparations were further concentrated using Amicon filters as described above. Mock (control) preparations were created from uninfected cells.

### 2.3. Binding of Flavivirus Preparations to Monoclonal Antibody-Coupled Microspheres for FlaviMIA

Each inactivated virus preparation was pre-titrated to determine optimal working dilution by flaviMIA testing of serial two-fold dilutions against known positive human sera and selecting the dilution giving maximum reactivity. Each virus preparation was diluted to 4 mL in MIA buffer (PBS + BSA (Sigma-Aldrich, USA) + ProClin300 (Sigma-Aldrich, USA) as a preservative) then mixed with its corresponding 6B6C-1-coupled beadset at 1:20. The virus–bead mixes were stored in light-resistant 4 mL bottles (Amber bottles, Nalgene, Rochester, NY, USA) which were incubated at room temperature on a shaker for several hours after which they were stored at 4 °C.

### 2.4. Reference Sera

Because infections by various viruses included in the flaviMIA occur infrequently (e.g., MVEV, ALFV, and STRV), characterized clinical reference sera are rare and often unavailable commercially.

Where possible, reference sera were sourced from IgM positive patients with RT-PCR-confirmed recent flavivirus infections who were willing to donate a large volume of sera for laboratory quality assurance purposes. When this was not possible, control sera were constructed by pooling reactive patient samples.

### 2.5. Flavivirus Screening Assays

**Figure 1:**
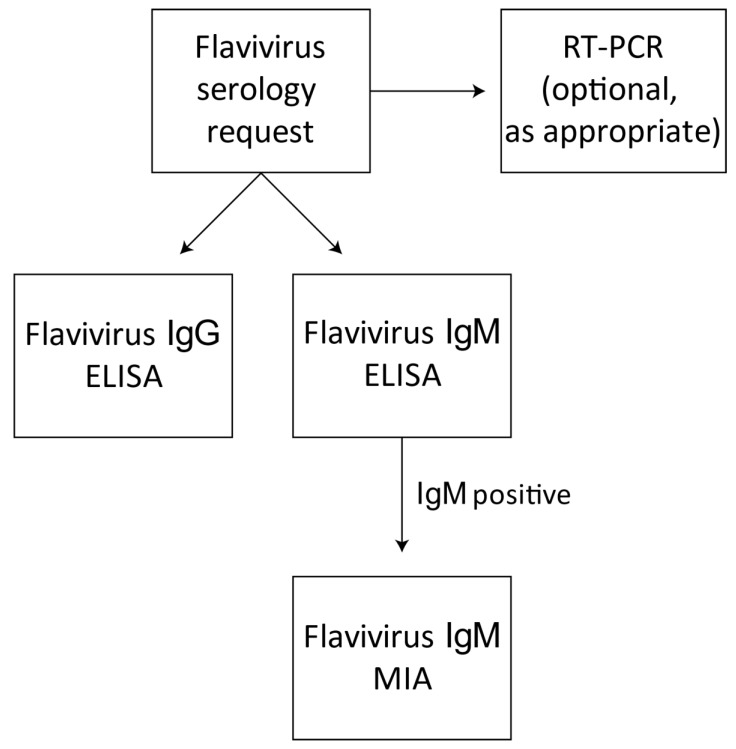
Test algorithm for flavivirus serology requests. When requested, sera are screened for flavivirus IgG and IgM by in-house ELISAs employing a pool of purified flaviviruses. Specimens determined to be reactive or equivocal in the flavivirus MAC-ELISA are then tested by the multiplexed flavivirus IgM typing microsphere immunoassay (flaviMIA). Specific RT-PCR tests may also be conducted on the sample as appropriate.

Patient serum samples referred to our laboratory from public hospitals or private diagnostic laboratories specifically for flavivirus serology were first screened for flavivirus IgG and IgM by in-house ELISAs (Figure 1). These ELISAs employ a pool of flaviviruses, as listed above, prepared using pre-titrated antigens at equivalent dilutions, combined into a 100 mL volume in 1× Milk Diluent (prepared from Milk Diluent/Blocking Solution Concentrate (Kirkegaard & Perry Laboratories, Gaithersburg, MD, USA)). A control pool was prepared from a mock antigen preparation from uninfected cells at the same combined dilution as the viral antigens. Specimens which exhibited flavivirus IgG reactivity in the absence of IgM by ELISA were reported as resulting from probable past flavivirus infections and a follow-up specimen was requested. Specimens giving positive results in the flavivirus IgM MAC-ELISA were then tested by flaviMIA to determine the specificity of the IgM, if possible (Figure 1).

Upon request, sera were also tested using two real-time RT-PCRs targeting the Asian lineage ZIKV non-structural protein 1 or envelope gene (Figure S1) [10]. A validated, previously described African lineage ZIKV real-time RT-PCR assay was available if travel history indicated a need. Both RT-PCRs were challenged using samples known to be positive for Chikungunya virus, each of the four DENV serotypes as well as a range of related sample types submitted for viral investigations of patients with related clinical presentations. Both tests were 100% specific. Briefly, 5 µL of RNA extracted from patient samples using the BioRobot^®^ Universal System and QIAamp One-For-All Nucleic Acid Kit, (Qiagen, Hilden, Germany) or EZ1 and Virus Mini Kit v2.0, (Qiagen, Hilden, Germany) were added to 15 µL RT-PCR reactions (SSIII Platinum One-Step qRT-PCR System, Invitrogen, Carlsbad, CA, USA; oligonucleotide sequences described previously [10]) and amplified using a RotorGene thermal cycler (Qiagen, Hilden, Germany). Samples were reverse transcribed for 5 min at 50 °C, incubated at 95 °C for 2 min, and then subjected to 40 cycles of 95 °C for 3 s and 60 °C for 30 s.

### 2.6. Flavivirus-Specific IgM Serology—FlaviMIA

Flavivirus IgM ELISA-positive serum specimens were treated with rheumatoid factor (RF) removal reagent (RF absorbent, Dade Behring, Deerfield, IL, USA) to remove potentially interfering IgG and rheumatoid factor. This was performed in a microtiter plate with 5 µL serum diluted 1:20 in 95 µL MIA buffer. RF removal reagent was added (100 µL) to each well, followed by incubation at room temperature for 15 min and a final dilution of 1:800 in MIA buffer.

The flaviMIA antibody binding reaction was then performed in 96-well filter plates (Millipore, USA), in wells pre-wetted with PBS-Tween. Each flavivirus and control beadset were further diluted 1:20 in MIA buffer in sufficient volume for the number of samples being tested. The bead mixture was added to the wells of the plate at 100 µL per well followed by washing in PBS-Tween using a Bio-Plex Pro II washer. Diluted sera were added to the plate, along with a specific positive control serum for each virus, and a negative serum in duplicate. The plate was incubated at room temperature with shaking for 45 min after which the washing was repeated. Phycoerythrin-labelled donkey anti-human IgM antibody (Jackson Immuno Research, West Grove, PA, USA) was diluted to previously-determined optimal concentration in MIA buffer and added to each well of the plate. Incubation and wash steps were repeated and the beads were resuspended in each well by addition of 150 µL MIA buffer.

**Figure 2:**
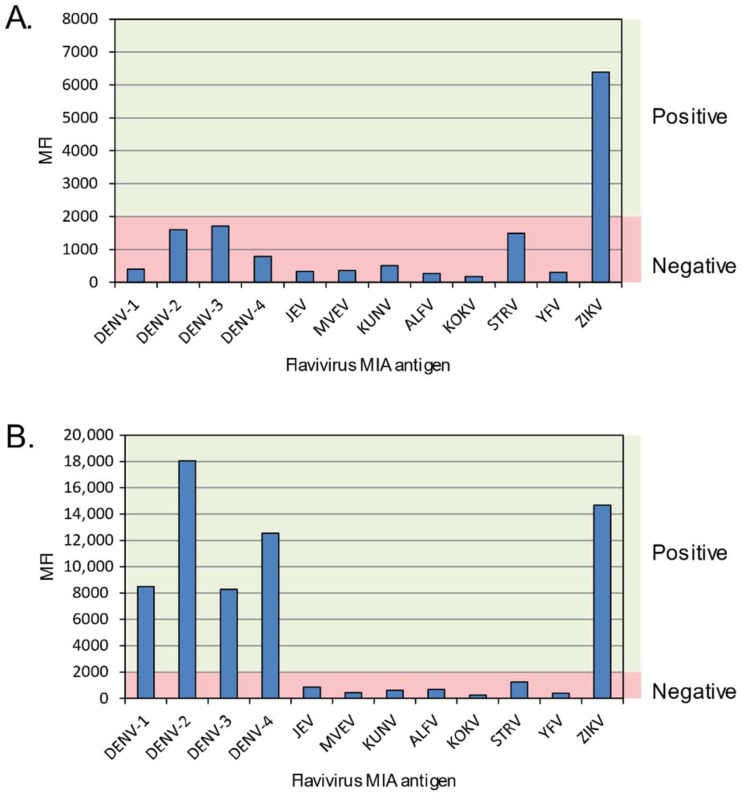
Multiplexed flavivirus IgM microsphere immunoassay (flaviMIA) result examples highlighting specific and cross-reactive IgM reactivity. (**A**) The intensity of the anti-ZIKV IgM signal, reported as mean fluorescence intensity (MFI, *y*-axis) compared to 11 other viruses (*x*-axis) used in the flaviMIA defined this sample from Patient Reference No. 7 as clearly containing ZIKV-specific IgM. (**B**) This ZIKV RNA positive representative sample from Patient Reference No. 11 was classified as having cross-reactive IgM towards both ZIKV and DENV. Red shading indicates the MFI signal range from a seronegative result; green shading indicates the region into which seropositive results fall. DENV, Dengue virus; JEV, Japanese encephalitis virus; MVEV, Murray Valley encephalitis virus; KUNV, Kunjin virus; ALFV, Alfuy virus; KOKV, Kokobera virus; STRV, Stratford virus; YFV, Yellow fever virus; ZIKV, Zika virus.

The plate was read on a Bio-Plex 200 instrument (Bio-Rad, USA), with 100 beads per well read in 100 µL of buffer using Bio-Plex Manager^®^ software (Version 6.0, Bio-Rad, Hercules, CA, USA). Results were exported to Microsoft Excel^®^ and presented graphically for easier determination of specificity and analysis. A mean fluorescent intensity (MFI) cut-off of 2000 units was assigned based on comparison to RT-PCR-confirmed dengue sera; values above 2000 were considered a positive result. Specificity determinations were based on the MFI result of one viral beadset preparation being 1.5 times higher than the MFI of any other beadset in the panel. Where a single virus-specific IgM could not be determined, i.e., if there was reactivity to two or more flaviviruses in the panel (excluding between dengue serotypes) the result was reported as “cross-reactive” (Figure 2). This cross-reactivity is known to occur in human sera following infection with a single virus, but the possibility of co-infection with at least two viruses is also known to occur and should be considered a possibility.

### 2.7. ZIKV PRNT

Because PRNT is considered a gold standard assay for confirmation of flavivirus antibodies, a ZIKV PRNT was conducted to interrogate ZIKV results from the ZIKV component of the flaviMIA. Sera were screened at a dilution of 1:10 after incubation at 37 °C for 90 min with an equivalent volume of diluted ZIKV MR766 whole virus antigen preparation. Serum from a confirmed ZIKV Asian lineage infected patient was used as a positive control and a pool of flavivirus negative sera was used as the negative control. ZIKV was pre-titrated to give approximately 75 plaques per well of a 6-well plate. After incubation, 100 µL of each serum/virus mix was inoculated in duplicate into 6-well plates seeded one day previously with Vero cells (ATCC^®^ CCL-81^TM^). Following virus absorption for 60 min, the cells were overlaid with a medium containing Gibco Opti-MEM (Thermo Fisher Scientific, Waltham, MA, USA), 3% foetal bovine serum (In Vitro Technologies, Noble Park North, Australia) and 1.5% carboxymethylcellulose (Sigma Aldrich, USA). The Vero cell plates were then incubated for seven days, after which the supernatant was removed and the cells washed with PBS and stained with naphthalene black stain (0.1% (*w*/*v*) naphthol Blue Black, 6% (*v*/*v*) glacial acetic acid, 1.36% sodium acetate). Plaques were counted and sera (at 1:10) demonstrating >90% plaque neutralization by comparison of plaque counts in duplicate “no antibody” control wells were reported as positive.

## 3. Results

### 3.1. FlaviMIA Validation

For the purpose of validation of the assay for ZIKV diagnosis, 38 acute phase sera and 25 convalescent phase (collected > 7 days after the first) sera from patients positive for ZIKV (RT-PCR or PRNT confirmed cases) but who have no serological evidence of previous flavivirus infection (primary ZIKV cases), 834 acute and convalescent-phase sera known to be positive for one of nine other flaviviruses and 70 flavivirus IgG and IgM screening ELISA negative sera were analyzed by flaviMIA (Table S1). The sensitivity was found to be 47.4% for acute sera and 100% for convalescent sera. Specificity of the assay was found to be 100%. The flaviMIA processing time varies depending on number of samples per run, but can be completed in as little as 2.5 h.

A strain from the ZIKV Asian lineage [15] was isolated from an infected traveler and used to create a purified whole virus antigen preparation to test representative sera to investigate whether both lineages performed similarly. Parallel testing of serum from six ZIKV-infected patients showed no significant titer differences between the African lineage and Asian lineage antigens (less than a two-fold difference in PRNT_90_ titer).

### 3.2. Analysis of Patient Samples

In responding to clinical and public health need, our laboratory has identified a total of 101 patients with flaviMIA results indicating specific IgM to ZIKV (Table 1). Of these, 43 were determined to be recent primary ZIKV infections (no serological evidence of previous flavivirus infection, Group 1) and 21 were recent secondary infections (flavivirus IgG or IgM in the acute sample suggestive of previous infection by, or vaccination with, another flavivirus, Group 2). A further 14 were determined as past ZIKV infections with persistent IgM as no change in antibody level was detected between acute and convalescent paired sera (Group 3). Two patients had serological results consistent with a recent dengue infection with a history of ZIKV infection, however concurrent infection with both viruses could not be excluded (Group 4). For 19 patients, ZIKV specific IgM was detected by flaviMIA and confirmed by PRNT, however, in the absence of a convalescent specimen collected ≥7 days post-onset, ZIKV infection could not be determined as having occurred recently (Group 5). A further two patients were found to be flaviMIA positive with ZIKV specific IgM but were neither RT-PCR nor PRNT positive and therefore could not be confirmed as ZIKV positive. One of these (Reference No. 56) was identified as a false ZIKV IgM positive as seroconversion was not demonstrated. The other (Reference No. 77) may have been collected too early in infection for neutralizing antibodies to be detected, but could not be excluded as a false positive.

### 3.3. Patients with Recent Primary ZIKV Infections

Of the 43 patients determined as having recent primary ZIKV infection, 23 (53.5%) had acute samples which were negative by flaviMIA while 20 (46.5%) were positive. All but one of the positive sera had specific ZIKV IgM. Twenty-eight (65.1%) of the 43 patients had a follow-up sample collected. In 27 patients, the flaviMIA was specific for ZIKV, while the other patient exhibited cross-reactivity to other flaviviruses (ZIKV positive, but reactivity also detected to DENV, JEV, ALFV and STRV).

### 3.4. Patients with Recent Secondary ZIKV Infections

Our laboratory also tested samples from 21 patients whose results indicated that infection with ZIKV was secondary to a previous flavivirus infection. Previous infection was determined by the presence of flavivirus IgG (detected by ELISA) concurrent with the detection of ZIKV RNA or in one case (Reference No. 24) Flavivirus IgG in the absence of IgM within five days of return from overseas travel. Of the 21 acute sera, nine (42.9%) were negative and the remainder were positive by flaviMIA. Six of the flaviMIA positive sera were ZIKV IgM specific, two were cross-reactive between ZIKV and at least one other virus, while the remainder had IgM that specifically reacted to either DENV-2 or DENV-4 (both suggesting previous infection with these dengue serotypes) and two to YFV (consistent with previous vaccination with YFV associated with travel to South America). Unfortunately, these prior infections could not be confirmed (by PRNT) owing to routinely insufficient sample volume, however pre-existing specific antibodies were confirmed by indirect fluorescent antibody test.

A convalescent serum sample was collected from 12 of the patients and each was tested in parallel with its paired acute serum to demonstrate change in antibody levels. Eight of the follow-up sera were positive and specific for ZIKV IgM including the acute sample with initial DENV-4 IgM (Reference No. 8), three from initially negative patients, three from the initially ZIKV-specific patients, and from one patient whose acute sample was YFV IgM reactive (Reference No. 83). Three further patients had cross-reactive IgM in the convalescent sample, two from initially negative patients (Reference No. 23 and 36) and one from the DENV-2 specific patient (Reference No. 11). One convalescent specimen (Reference No. 10) was DENV-2 specific by flaviMIA with ZIKV infection clearly demonstrated by both RNA detection and PRNT. Co-infection of ZIKV and DENV in this patient cannot be excluded, although DENV RNA was not detected.

### 3.5. Patients with Past ZIKV Infection

Past ZIKV infection was demonstrated in 14 patients by parallel testing demonstrating stable antibody levels between the samples. One of the 14 acute samples had cross-reactive IgM (Reference No. 90) while the remainder were ZIKV IgM specific. All convalescent sera were ZIKV-IgM specific and all were confirmed by PRNT.

### 3.6. Patients with ZIKV/DENV IgM

Patient Reference No. 17 had a single sample which was DENV-1 RNA positive, but the flaviMIA result was ZIKV IgM specific. The sample was also positive by ZIKV PRNT, suggesting previous infection or exposure to ZIKV. Balmaseda et al. reported DENV-1 to be the most common DENV to cross-react with ZIKV [16].

Patient Reference No. 22 was found to have ZIKV-specific IgM and ZIKV neutralizing antibodies in the acute sample, while the flaviMIA result on the convalescent sample showed cross-reactive IgM between ZIKV and DENV. This result is suggestive of dengue infection secondary to an earlier infection or exposure to ZIKV.

## 4. Discussion

Huge numbers of suspected ZIKV infections were recorded across the Americas in 2015 and 2016 but most were not confirmed by laboratory methods. This and other regions have co-circulating viruses which can produce a clinically indistinguishable initial picture to that from ZIKV infection. Laboratory methods are the only way to ensure accurate diagnoses. With the discovery of congenital Zika virus syndrome, robust diagnosis to support patient prognosis has become more urgent however, definitive results require a complex and resource intensive process. Molecular methods used on serum samples rapidly, sensitively and accurately identify ZIKV RNA but suffer from the short window of detection using sera. Whole blood and urine may extend this window over testing of sera alone [17,18].

Other researchers have described new serological tests incorporating panels of flaviviruses but these usually include fewer virus targets than we have, are region-specific, or use recombinant rather than whole-virus antigens [16,19,20,21,22]. One MIA method included an avidity determination step to reduce weaker, presumably less specific, false positive reactions, to good effect in known ZIKV or DENV infected returning travelers or among Swedish blood donors [23]. However, this method yielded low MFI values compared to the flaviMIA. Avidity testing also aided a new microarray platform which performed better on convalescent than acute samples [22]. New and traditional culture-based methods have also been described, but results can take days rather than hours, may remain unclear without additional testing and generally only account for a subset of flaviviruses [24]. Each of these approaches has advantages and disadvantages but it is important to include a well characterized specimen panel assembled from travelers with a potentially complex history of past flavivirus exposures.

Paired sera, collected two weeks apart, are essential for antibody-based identification of virus infection and serology is important to support the diagnosis of ZIKV infection. Despite only 60.6% of our sera being paired, the addition of a convalescent sample frequently permitted clarification of an initially unclear result as when IgM persisted from a past flavivirus infection or vaccination. This has been reported by others [25]. Hence, an acute and a convalescent serum pair are desirable to measure a seroconversion, a significant rise in specific antibody titer or a drop in titer against an unexpected virus. Reactive flavivirus serology results on single samples cannot be reliably interpreted [24]. For some patients with a clinical picture of ZIKV but a negative or equivocal or cross-reactive result from an acute serum, collecting a convalescent serum can mean prolonging the final result. This is especially unsatisfactory for pregnant women. Alternative rapid methods are needed, or testing of whole blood or urine samples using RT-PCR, which are known to contain signs of virus for longer than serum.

In our study, a combination of RT-PCR and flavivirus serology identified and confirmed the presence of ZIKV infection in 99 suspected cases. By combining molecular and serological techniques, we could differentiate primary from secondary infections and recent from past infections in most cases as described by others [26]. The difficulties of flavivirus laboratory investigations were exemplified by results from two patients with evidence of past exposure to DENV (they were defined as secondary flavivirus infections because of the presence of DENV IgG who were producing IgM which was specific to DENV but not to the RT-PCR-positive current ZIKV infection. This may represent a new DENV co-infection but is more likely to be evidence of “original antigenic sin” [27,28]; the anamnestic production of antibody to a past (DENV) infection, resulting from a new yet different (ZIKV) infection prior to the development of specific antibodies to the more recently encountered virus. Either outcome would be unsurprising in travelers from geographic regions where a range of arboviruses may co-circulate. Possible ZIKV co-infections are being reported more often as investigations broaden and more inclusive testing protocols are employed [29,30,31]. A multifaceted approach to testing, rather than reliance solely on RT-PCR or serology, would ideally improve results [32].

Patients originating from some areas of the world may have been exposed to multiple flaviviruses over a lifetime. Among primary ZIKV infections, we found antibody reactivity by flaviMIA was usually specific when follow-up samples were available and collected within a relevant time-frame from onset of disease. In the case of secondary infections however, results were often less clear, with the earliest detected antibody often directed towards a presumed earlier virus, or to prior YFV vaccination.

The flaviMIA is a specialized technique requiring an experienced laboratory. The use of a multi-target antibody testing protocol such as this for screening samples from patients with suspected flavivirus infections benefits from including viral targets most appropriate to a patient’s exposure history [13].

The results of our study suggest that all patients with suspected ZIKV infection should have a submitted specimen tested initially by RT-PCR (see Supplementary Materials). Where RNA is detected, ZIKV infection is confirmed without further testing. However, where specimens are negative by RT-PCR (due to late collection, poor sample quality or low viral load), then the flaviMIA is useful to determine presence and specificity of flavivirus IgM. In some cases, PRNT may be required to confirm ZIKV infection. We suggest that a minimum of 1 mL of serum be supplied for testing to ensure sufficient is available for all tests that may be required.

Our in-house flaviMIA protocol affords broad concurrent flavivirus serodiagnostic capacity. In our professional laboratory setting, it proved easy to use, was amenable to high throughput testing, had a short turnaround time and generated reproducible results. The flaviMIA was rapid in both analysis time and in interpretation of results. However, our results highlight the complexities of flavivirus diagnostic serology, which can be confounded by several factors including the presence of cross-reactive antibodies and high titers of specific antibody to antigenically different viruses that have resulted from recent or concurrent infection, vaccination or because of an anamnestic response triggered by a new ZIKV infection.

## Figures and Tables

**Table 1:** Laboratory data for 101 patients with flaviMIA results indicating specific IgM to ZIKV.

Group Reference Number	Laboratory Reference No.	Sex	Age	Flavivirus IgG ELISA Result	Flavivirus IgG ELISA Results	FlaviMIA Result	FlaviMIA Result	SI	RT-PCR Result	PRNT Result	Year/Travel Region
				1st Sample	2nd Sample	1st Sample	2nd Sample				
**Group 1: recent ZIKV infection—primary**											
#1	1	M	40	Negative	Positive	Negative	Positive (ZIKV specific)	19	Not detected	Positive	2013 Thailand
#2	2	M	34	Negative	Positive	Negative	Positive (ZIKV specific)	14	Not detected	Positive	2013 Vietnam
#3	3	M	30	Negative	nss	Negative	nss	n/a	ZIKV RNA detected	Insufficient	2014 Cook Islands
#4	7	M	58	Negative	Positive	Negative	Positive (ZIKV specific)	6	ZIKV RNA detected	Positive	2014 Cook Islands
#5	9	M	32	Negative	nss	Negative	nss	n/a	ZIKV RNA detected	Negative	2014 Cook Islands
#6	12	M	38	Negative	nss	Negative	nss	n/a	ZIKV RNA detected	Insufficient	2014 Cook Islands
#7	13	M	79	Negative	nss	Negative	nss	n/a	ZIKV RNA detected	Insufficient	2014 Cook Islands
#8	14	M	24	Negative	Negative	Negative	Positive (ZIKV specific)	4	ZIKV RNA detected	Negative	2014 Cook Islands
#9	15	F	7	Negative	Positive	Negative	Positive (ZIKV specific)	35	Not detected	Positive	2014 Country of travel not identified
#10	16	F	37	Negative	Positive	Positive (ZIKV specific)	Positive (ZIKV specific)	15	Not detected	Positive	2014 Country of travel not identified
#11	19	F	42	Negative	Positive	Negative	Positive (cross-reactive)	19	ZIKV RNA detected	Positive	2014 Vanuatu
#12	20	F	43	Negative	Positive	Negative	Positive (ZIKV specific)	16	Not detected	Positive	2015 Bali
#13	26	M	48	Negative	nss	Negative	nss	n/a	ZIKV RNA detected	Negative	2015 Solomon Islands
#14	27	M	45	Negative	nss	Negative	nss	n/a	ZIKV RNA detected	Negative	2015 Solomon Islands
#15	29	M	33	Negative	Positive	Positive (ZIKV specific)	Positive (ZIKV specific)	15	ZIKV RNA detected	Positive	2015 Solomon Islands
#16	30	M	52	Negative	nss	Positive (ZIKV specific)	nss	n/a	Not detected	Positive	2015 Solomon Islands
#17	32	M	29	Negative	Positive	Negative	Positive (ZIKV specific)	91	Not detected	Positive	2015 Solomon Islands
#18	33	F	22	Negative	Positive	Negative	Positive (ZIKV specific)	10	ZIKV RNA detected	Positive	2015 Vanuatu
#19	34	F	33	Negative	nss	Positive (ZIKV specific)	nss	n/a	ZIKV RNA detected	Positive	2015 Vanuatu
#20	47	F	30	Negative	Positive	Positive (ZIKV specific)	Positive (ZIKV specific)	14	ZIKV RNA detected	Positive	2016 Fiji
#21	52	F	22	Negative	Positive	Positive (ZIKV specific)	Positive (ZIKV specific)	17	Not detected	Positive	2016 Fiji
#22	53	F	62	Negative	Positive	Positive (ZIKV specific)	Positive (ZIKV specific)	16	Not detected	Positive	2016 Fiji
#23	57	F	17	Negative	Positive	Positive (ZIKV specific)	Positive (ZIKV specific)	24	Not detected	Positive	2016 Fiji/Samoa
#24	60	M	50	Negative	nss	Negative	nss	n/a	ZIKV RNA detected	Insufficient	2016 Guyana
#25	61	M	30	Negative	Positive	Positive (ZIKV specific)	Positive (ZIKV specific)	26	ZIKV RNA detected	Positive	2016 Jamaica
#26	64	F	33	Negative	nss	Positive (ZIKV specific)	nss	n/a	ZIKV RNA detected	Negative	2016 Mexico
#27	65	F	33	Negative	Positive	Negative	Positive (ZIKV specific)	14	Not detected	Positive	2016 Mexico
#28	68	M	37	Negative	Positive	Positive (ZIKV specific)	Positive (ZIKV specific)	8	Not detected	Positive	2016 Mexico
#29	69	F	42	Negative	Positive	Positive (ZIKV specific)	Positive (ZIKV specific)	21	Not detected	Positive	2016 Mexico
#30	71	F	21	Negative	nss	Positive (ZIKV specific)	nss	n/a	Not detected	Positive	2016 Nicaragua
#31	74	F	28	Negative	Positive	Negative	Positive (ZIKV specific)	16	ZIKV RNA detected	Positive	2016 Samoa
#32	76	F	2	Negative	nss	Positive (ZIKV specific)	nss	n/a	ZIKV RNA detected	Positive	2016 Samoa
#33	81	F	28	Negative	Positive	Positive (ZIKV specific)	Positive (ZIKV specific)	15	Not detected	Positive	2016 Solomon Is
#34	84	F	43	Negative	Positive	Positive (ZIKV specific)	Positive (ZIKV specific)	14	Not detected	Positive	2016 Thailand
#35	86	F	37	Negative	nss	Negative	Positive (ZIKV specific)	46	ZIKV RNA detected	Positive	2016 Tonga
#36	88	F	52	Negative	nss	Negative	nss	n/a	ZIKV RNA detected	Negative	2016 Tonga
#37	89	F	23	Negative	Positive	Positive (cross-reactive)	Positive (ZIKV specific)	14	Not detected	Positive	2016 Tonga
#38	92	M	41	Negative	Negative	Positive (ZIKV specific)	Positive (ZIKV specific)	11	Not detected	Positive	2016 Tonga
#39	94	M	43	Negative	Positive	Positive (ZIKV specific)	Positive (ZIKV specific)	94	ZIKV RNA detected	Positive	2016 Vanuatu/Fiji
#40	95	F	56	Negative	nss	Negative	nss	n/a	ZIKV RNA detected	Negative	2016 Vietnam
#41	97	F	71	Negative	Positive	Positive (ZIKV specific)	Positive (ZIKV specific)	12	Not detected	Positive	2017 Country of travel not identified
#42	99	F	49	Negative	nss	Negative	nss	n/a	Not detected	Positive	2017 Cuba
#43	100	M	33	Negative	Positive	Negative	Positive (ZIKV specific)	20	ZIKV RNA detected	Positive	2017 Cuba
**Group 2: recent ZIKV infection—secondary**											
#1	4	F	42	Positive	nss	Positive (cross-reactive)	nss	n/a	ZIKV RNA detected	Positive	2014 Cook Islands
#2	5	M	30	Positive	nss	Positive (ZIKV specific)	nss	n/a	ZIKV RNA detected	Negative	2014 Cook Islands
#3	6	F	43	Positive	nss	Positive (cross-reactive)	nss	n/a	ZIKV RNA detected	Insufficient	2014 Cook Islands
#4	8	F	65	Positive	Positive	Positive (DENV-4 specific)	Positive (ZIKV specific)	23	ZIKV RNA detected	Positive	2014 Cook Islands
#5	10	F	38	Positive	Positive	Negative	Positive (DENV-2 specific)	20	ZIKV RNA detected	Positive	2014 Cook Islands
#6	11	M	31	Positive	Positive	Positive (DENV-2 specific)	Positive (cross-reactive)	23	ZIKV RNA detected	Positive	2014 Cook Islands
#7	23	F	51	Positive	Positive	Negative	Positive (cross-reactive)	30	ZIKV RNA detected	Positive	2015 El Salvador
#8	24	M	55	Positive	Positive	Negative	Positive (ZIKV specific)	25	Not detected	Positive	2015 Samoa
#9	25	M	46	Positive	nss	Negative	nss	n/a	ZIKV RNA detected	Insufficient	2015 Solomon Islands
#10	28	F	26	Positive	nss	Negative	nss	n/a	ZIKV RNA detected	Positive	2015 Solomon Islands
#11	36	M	54	Positive	Positive	Negative	Positive (cross-reactive)	13	ZIKV RNA detected	Positive	2016 Bali
#12	38	M	25	Negative	nss	Positive (YFV specific)	nss	n/a	ZIKV RNA detected	Negative	2016 Colombia
#13	45	M	42	Positive	Positive	Positive (ZIKV specific)	Positive (ZIKV specific)	15	ZIKV RNA detected	Positive	2016 El Salvador
#14	46	F	45	Positive	Positive	Negative	Positive (ZIKV specific)	13	ZIKV RNA detected	Positive	2016 Fiji
#15	62	F	25	Positive	Positive	Positive (ZIKV specific)	Positive (ZIKV specific)	14	ZIKV RNA detected	Positive	2016 Mexico
#16	63	M	34	Positive	nss	Positive (ZIKV specific)	nss	n/a	ZIKV RNA detected	Negative	2016 Mexico
#17	75	M	52	Positive	nss	Positive (ZIKV specific)	nss	n/a	ZIKV RNA detected	Positive	2016 Samoa
#18	82	F	60	Positive	Positive	Negative	Positive (ZIKV specific)	22	ZIKV RNA detected	Positive	2016 Solomon Islands
#19	83	F	30	Negative	Positive	Positive (YFV specific)	Positive (ZIKV specific)	18	ZIKV RNA detected	Positive	2016 Thailand
#20	85	F	71	Positive	Positive	Positive (ZIKV specific)	Positive (ZIKV specific)	13	ZIKV RNA detected	Positive	2016 Tonga
#21	87	M	34	Positive	nss	Negative	nss	n/a	ZIKV RNA detected	Insufficient	2016 Tonga
**Group 3: patients with past ZIKV infection**											
#1	18	M	77	Positive	Positive	Positive (ZIKV specific)	Positive (ZIKV specific)	11	Not detected	Positive	2014 Papua New Guinea
#2	21	M	43	Positive	Positive	Positive (ZIKV specific)	Positive (ZIKV specific)	30	Not tested	Positive	2015 Brazil
#3	37	F	68	Positive	Positive	Positive (ZIKV specific)	Positive (ZIKV specific)	9	Not tested	Positive	2016 Caribbean
#4	39	F	23	Positive	Positive	Positive (ZIKV specific)	Positive (ZIKV specific)	26	Not detected	Positive	2016 Colombia
#5	40	M	35	Positive	Positive	Positive (ZIKV specific)	Positive (ZIKV specific)	22	Not detected	Positive	2016 Country of travel not identified
#6	41	F	39	Positive	Positive	Positive (ZIKV specific)	Positive (ZIKV specific)	14	Not detected	Positive	2016 Country of travel not identified
#7	43	F	61	Positive	Positive	Positive (ZIKV specific)	Positive (ZIKV specific)	17	Not detected	Positive	2016 Curacao
#8	51	F	23	Positive	Positive	Positive (ZIKV specific)	Positive (ZIKV specific)	17	Not detected	Positive	2016 Fiji
#9	67	M	48	Positive	Positive	Positive (ZIKV specific)	Positive (ZIKV specific)	28	Not detected	Positive	2016 Mexico
#10	72	M	88	Positive	Positive	Positive (ZIKV specific)	Positive (ZIKV specific)	9	Not detected	Positive	2016 No recent travel
#11	73	M	82	Positive	Positive	Positive (ZIKV specific)	Positive (ZIKV specific)	20	Not detected	Positive	2016 No recent travel
#12	80	M	73	Positive	Positive	Positive (ZIKV specific)	Positive (ZIKV specific)	36	Not detected	Positive	2016 Solomon Is
#13	90	M	27	Positive	Positive	Positive (cross-reactive)	Positive (ZIKV specific)	14	Not detected	Positive	2016 Tonga
#14	91	F	27	Positive	Positive	Positive (ZIKV specific)	Positive (ZIKV specific)	10	Not detected	Positive	2016 Tonga
**Group 4: patients with ZIKV/DENV IgM**											
#1	17	F	43	Positive	nss	Positive (ZIKV specific)	nss	n/a	Not detected	Positive	2014 Maldives
#2	22	M	78	Positive	Positive	Positive (ZIKV specific)	Positive (ZIKV/DENV)	16	Not tested	Positive	2015 Burma
**Group 5: patients with recent or past ZIKV infection**											
#1	31	M	37	Positive	nss	Positive (ZIKV specific)	nss	n/a	Not detected	Positive	2015 Solomon Islands
#2	35	F	34	Positive	nss	Positive (ZIKV specific)	nss	n/a	Not tested	Positive	2015 Vanuatu
#3	42	M	31	Positive	nss	Positive (ZIKV specific)	nss	n/a	Not detected	Positive	2016 Country of travel not identified
#4	44	F	57	Positive	Positive	Positive (ZIKV specific)	Positive (ZIKV specific)	6	Not detected	Positive	2016 Dominican Republic
#5	48	F	25	Positive	nss	Positive (ZIKV specific)	nss	n/a	Not detected	Positive	2016 Fiji
#6	49	M	56	Positive	nss	Positive (ZIKV specific)	nss	n/a	Not detected	Positive	2016 Fiji
#7	50	F	41	Positive	nss	Positive (ZIKV specific)	nss	n/a	Not detected	Positive	2016 Fiji
#8	54	M	12	Positive	nss	Positive (ZIKV specific)	nss	n/a	Not detected	Positive	2016 Fiji
#9	55	F	31	Positive	nss	Positive (ZIKV specific)	nss	n/a	Not tested	Positive	2016 Fiji
#10	58	M	54	Positive	nss	Positive (ZIKV specific)	nss	n/a	Not detected	Positive	2016 Fiji/Tonga
#11	59	F	30	Positive	nss	Positive (ZIKV specific)	nss	n/a	Not tested	Positive	2016 Guatemala/Belize
#12	66	F	25	Positive	nss	Positive (ZIKV specific)	nss	n/a	Not detected	Positive	2016 Mexico
#13	70	F	36	Positive	nss	Positive (ZIKV specific)	nss	n/a	Not detected	Positive	2016 Mexico
#14	78	M	73	Positive	nss	Positive (ZIKV specific)	nss	n/a	Not detected	Positive	2016 Samoa
#15	79	F	27	Positive	nss	Positive (ZIKV specific)	nss	n/a	Not detected	Positive	2016 Solomon Is
#16	93	F	31	Positive	nss	Positive (ZIKV specific)	nss	n/a	Not detected	Positive	2016 Tonga
#17	96	M	32	Positive	nss	Positive (ZIKV specific)	nss	n/a	Not detected	Positive	2016 Vietnam/Myanmar
#18	98	F	20	Positive	nss	Positive (ZIKV specific)	nss	n/a	Not detected	Positive	2017 Country of travel not identified
#19	101	F	36	Positive	nss	Positive (ZIKV specific)	nss	n/a	Not tested	Positive	2017 Fiji
**Group 6: patients with unconfirmed ZIKV infection/false positives**											
#1	56	M	16	Negative	Negative	Positive (ZIKV specific)	Positive (ZIKV specific)	12	Not detected	Negative	2016 Fiji
#2	77	F	52	Negative	nss	Positive (ZIKV specific)	nss	n/a	Not detected	Negative	2016 Samoa

SI, sampling interval; nss, no specimen submitted; n/a, not applicable.

## Supplementary Materials

The following are available online at http://www.mdpi.com/1999-4915/10/5/253/s1, Figure S1: Comprehensive testing algorithm for ZIKV requests, Table S1: Validation of flaviMIA.
