# Supplementary material for: Detection of Specific ZIKV IgM in Travelers Using a Multiplexed Flavivirus Microsphere Immunoassay
Source: Viruses. 2018 May 12;10(5):253. doi: 10.3390/v10050253 (PMC5977246; doi:10.3390/v10050253)
Supplement: Supplementary file 1 [file viruses-10-00253-s001.pdf]

Supplementary Materials:

Validation of flaviMIA

A total of 897 positive samples and 70 negative sera were selected as a test validation panel.

Well characterised sera from RT-PCR-confirmed primary cases were selected. Because the specificity of IgM does not always reflect the current infecting serotype in secondary dengue cases, presence of RNA was used for sample selection rather than IgM detection.

Acute and convalescent sera are described separately. Sensitivity and specificity data for each component of the assay are listed in Supplementary Table 1. After optimisation, an arbitrary cut-off of 2000 MFI was chosen and sensitivity and specificity of the assay were compared to the PCR results. A ratio of 1.5 was set to determine the specificity of the antibody detected i.e. where the MFI output for antibody reactivity to one virus was 1.5 or more times higher than that signal to any other viruses in the panel, the signal was described as specific antibody reactivity to the first virus. This factor allowed 33.7% of all sera to be correctly assigned to a virus species (i.e. dengue serotype, ZIKV etc.), and a further 19.6% to be correctly assigned to a virus group (e.g. to DENV). The remainder of the sera were described as Flavivirus cross-reactive, or negative.

Table S1. Description of samples used to validate the flaviMIA.

Sera	No. Positive sera	Reference assay	flaviMIA Sensitivity	flaviMIA Specificity
DENV-1 Acute	270	PCR	44.8	97.1
DENV-1	42	PCR[1]	100.0	97.1
DENV-2 Acute	161	PCR	49.1	97.1
DENV-2	31	PCR[1]	100.0	97.1
DENV-3 Acute	106	PCR	47.2	98.6
DENV-3	21	PCR[1]	95.2	98.6
DENV-4 Acute	59	PCR	45.8	95.7
DENV-4	16	PCR[1]	93.8	95.7
JEV	29	UCHI/HAI	93.1	100.0
MVEV	3	PCR, clinical	66.7	100.0
KUNV	41	UCHI/HAI	70.7	100.0
ALFV	0	Samples not available	Samples not	Samples not
KOKV	27	UCHI/HAI	81.5	100.0
STRV	0	Samples not available	Samples not	Samples not
YFV	28	Post-vaccination sera,	92.9	98.6
ZIKV Acute	38	PCR, PRNT	47.4	100.0
ZIKV	25	PCR*, PRNT	100.0	100.0

[1]RNA detected in acute sample from same patient; UCHI – ultracentrifugation-hemagglutination inhibition; HAI – hemagglutination inhibition assay

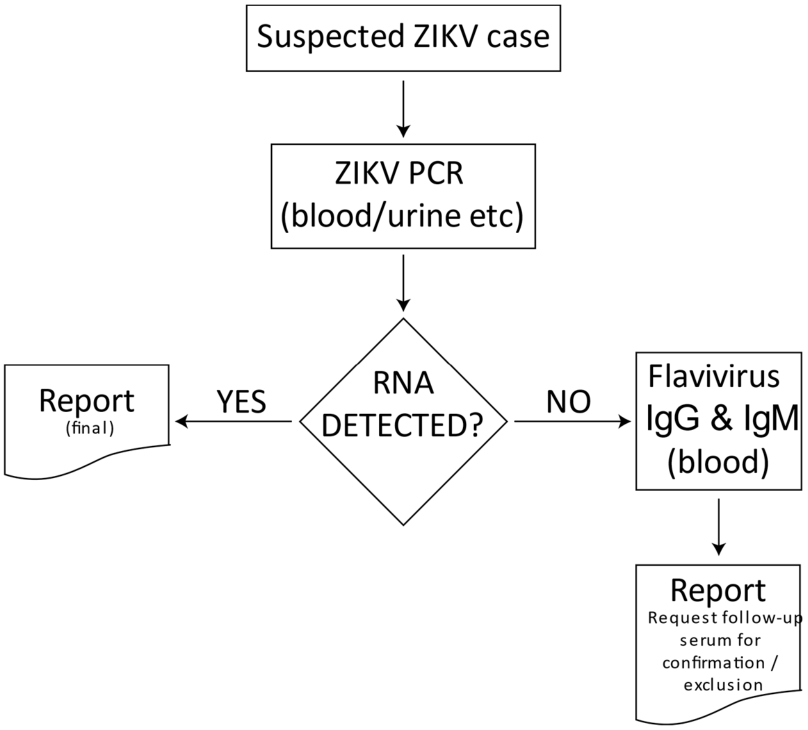

Figure S1. Comprehensive testing algorithm for ZIKV requests.

When specifically requested, a nucleic acid extract from sera is screened for ZIKV RNA using two previously described Asian-lineage specific real-time RT-PCR methods.{6095}

Specimens from which ZIKV RNA is not detected are examined for the presence of flavivirus antibodies following the process outlined in Figure 1.
